# Indirect somatic embryogenesis of *Agave maximiliana* Baker

**DOI:** 10.3389/fpls.2025.1648362

**Published:** 2025-08-28

**Authors:** Lourdes Delgado-Aceves, Santiago Corona, José Juvencio Castañeda-Nava, José Manuel Rodríguez-Domínguez, Antonia Gutiérrez-Mora

**Affiliations:** ^1^ Unidad de Biotecnología Vegetal, Centro de Investigación y Asistencia en Tecnología y Diseño del Estado de Jalisco, Zapopan, Jalisco, Mexico; ^2^ Departamento de Botánica y Zoología, Centro Universitario de Ciencias Biológicas y Agropecuarias, Universidad de Guadalajara, Zapopan, Jalisco, Mexico

**Keywords:** zygotic embryo, viability, callus, somatic embryo, auxins, Raicilla

## Abstract

*Agave maximiliana* Baker is a key species in the production of the traditional mexican beverage “Raicilla”. However, its slow reproductive cycle, lack of vegetative propagation, and increased commercial exploitation pose a threat to the conservation of its natural populations. This study presents, for the first time, a protocol for indirect somatic embryogenesis from zygotic embryos of *A. maximiliana*, as a biotechnological strategy for its mass propagation and conservation. Mature seeds were collected, and their viability was assessed using tetrazolium staining, revealing a significant loss of viability in seeds stored for more than two years. Embryonic axes were cultured on Murashige and Skoog medium supplemented with different concentrations of 2,4-D or picloram in combination with BAP. The highest embryogenic callus formation rates (up to 90%) were obtained with the treatment consisting of 36.24 µM 2,4-D, 33.12 µM picloram, and 4.44 µM BAP, resulting in an embryo-forming capacity (EFC) of 20%. Histological analyses confirmed the presence of proembryogenic cell masses and somatic embryos at different developmental stages, indicating an asynchronous regenerative system. The conversion rate of embryos into viable plantlets reached up to 90%, although some abnormalities were observed, associated with high auxin concentrations. This protocol represents a valuable tool for the conservation, reforestation, and sustainable production of this endemic and economically important species.

## 1 Introduction

*Agave maximiliana* Baker (commonly known as “Lechuguilla”) is a species of the Asparagaceae family with a geographic distribution in western Mexico. It primarily grows on rocky slopes within tropical deciduous oak and pine-oak forests, at elevations between 1500 and 2000 masl (Vázquez-García et al., 2007; Santacruz-Ruvalcaba et al., 2022). Due to its high sugar content and its traditional management practices, *A. maximiliana* is used in the production of the distilled beverage “Raicilla.” This beverage was recently granted a designation of appellation origin (Diario Oficial de la Federación, 2019), covering 16 municipalities in the state of Jalisco—Atengo, Chiquilistlán, Juchitlán, Tecolotlán, Tenamaxtlán, Puerto Vallarta, Cabo Corrientes, Tomatlán, Atenguillo, Ayutla, Cuautla, Guachinango, Mascota, Mixtlán, San Sebastián del Oeste, and Talpa de Allende—and one in the state of Nayarit: Bahía de Banderas.

Several *Agave* species found in the region have been used for Raicilla production, including *A*. *inaequidens, A. valenciana* (in the mountainous areas), and *A. angustifolia* and *A. rhodacantha* (in the coastal regions of Jalisco and Nayarit). However, *A. maximiliana* currently stands out as the principal species exploited for commercial production (Hernández-Cuevas et al., 2023). The recent rise in Raicilla’s popularity has led to increased consumption and the establishment of extensive agave plantations, often replacing traditional agroforestry systems and contributing to the overharvesting of wild populations (Hernández-Cuevas et al., 2023; Delgado-Aceves et al., 2024a). One example is *A. valenciana*, a cohabiting species with *A. maximiliana*, which has recently been classified as critically endangered by The IUCN Red List of Threatened Species™ due to a sharp decline in its natural populations (Torres-García et al., 2018).

In *A. maximiliana*, propagation is limited to seed germination from mature fruits, which develop approximately four months after pollination. The flowering stalk (scape) typically emerges after seven years (in early maturing individuals at 1500 masl) and up to ten years in others (personal communication, december 2023). This reproductive limitation, combined with the absence of asexual propagation (e.g., bulbils or rhizomatous shoots), makes the species highly vulnerable to degradation of its natural populations. Additionally, seeds produced on the scape are exposed to human extraction, natural degradation, predation by animals, and other biotic and abiotic factors, further reducing the species’ survival rate. Despite the progressive agroindustrial exploitation of *A. maximiliana*, studies have noted that plantations from various producers still maintain notable levels of genetic diversity. Morphological variability in monoculture plantations reflects the species’ recent domestication history. However, other commercially cultivated *Agave* species, such as *A. tequilana* Weber cv. Azul, *A. cupreata*, and *A. potatorum*, have undergone artificial selection pressures that have led to reduced genetic diversity, altered genetic structure, and signs of inbreeding (Cabrera-Toledo et al., 2020). To conserve the genetic variability of *A. maximiliana* without compromising Raicilla production, agroforestry practices should be promoted, and monoculture systems must be modified through agroecological approaches (Torres-García et al., 2019). These should be coupled with long-term conservation strategies using biotechnological tools (Hernández-Cuevas et al., 2023; Delgado-Aceves et al., 2024b). *In vitro* plant tissue culture can serve as a key component in integrated management strategies for threatened and endangered species, as it enables large-scale propagation of individuals for reforestation and commercial use, without further endangering wild populations (Chávez-Ortiz et al., 2021).

Recent reviews on somatic embryogenesis-based regeneration in *Agave* species highlight the development of micropropagation protocols in more than ten species of commercial interest. These include *A. victoria-reginae* (Rodríguez-Garay et al., 1996), *A. sisalana* (Nikam et al., 2003), *A. vera-cruz* Mill (Tejavathi et al., 2007), *A. tequilana* var. Azul (Portillo et al., 2007), *A. angustifolia* (Arzate-Fernández et al., 2016), *A. fourcroydes* (Monja-Mio and Robert, 2013), *A. americana* (Naziri et al., 2019), *A. marmorata* (Martínez-Martínez et al., 2021), *A. tequilana* cv. ‘Chato’ (Delgado-Aceves et al., 2022), and *A. salmiana* (Ángeles-Vázquez et al., 2023). These biotechnological advancements have been applied to optimize the production of diverse *Agave*-based products, including distilled and fermented beverages, fibers, ornamental and medicinal uses, as well as derivatives like syrups and agave honey (Bautista-Montes et al., 2022). However, a deeper understanding of the hormonal control mechanisms involved in these regeneration protocols is essential, as *Agave* species exhibit differential responses (Nic-Can and Loyola-Vargas, 2016). Somatic embryogenesis in *Agave* species has been most successfully induced using synthetic auxins such as 2,4-dichlorophenoxyacetic acid (2,4-D) and picloram, often in combination with cytokinins like kinetin or benzylaminopurine (BAP). These plant growth regulators play a key role in callus induction, maintenance, and embryo formation. For instance, 2,4-D has been widely used to initiate embryogenic calli in *A. tequilana* (Portillo et al., 2007) *A. angustifolia* (Arzate-Fernández et al., 2016) and *A. salmiana* (Ángeles-Vázquez et al., 2023, while picloram has shown higher efficiency *A. fourcroydes* and cultivars of *A. tequilana* (Delgado-Aceves et al., 2021). The concentration, duration of exposure, and interaction between auxins and cytokinins significantly affect the embryogenic response and are critical factors for successful large-scale propagation (Nikam et al., 2003; Tejavathi et al., 2007; Rodríguez-Garay, 2016).

In this study, we report for the first time the induction of somatic embryos in *A. maximiliana* through indirect somatic embryogenesis. This could support the sustainable supply of planting material for Raicilla production while preserving genetic diversity, contribute to the species medium- and long-term conservation, and generate large-scale plant material for reforestation and ecological restoration efforts.

## 2 Materials and methods

### 2.1 Seed collection

Mature fruits were collected in May 2023 and 2024 from five *Agave maximiliana* individuals aged 7 to 8 years, growing in a natural population located in San Jose del Mosco, Mascota, Jalisco, Mexico (20°33’10.7”N, 104°54’02.0”W; 20.552984, -104.900551). To assess seed viability, additional seeds from previous years’ collections—donated by local producers from the same region—were also used.

### 2.2 Seed germination and viability assessment

Germination tests were performed in accordance with the guidelines of the International Seed Testing Association (International Seed Testing Association, 2021). Given the limited availability of viable (black-colored) seeds, the number of seeds per experimental unit was adjusted accordingly. For each year, four replicates of 50 randomly selected black seeds were used. Seeds were imbibed in distilled water for 24 hours prior to sowing. Following imbibition, seeds were placed above moist paper towels within plastic containers. Germination was assessed ten days after establishment, and seeds were considered germinated when the radicle extended at least 5 mm beyond the seed coat. All tests were conducted under controlled conditions at 25 °C in complete darkness. Germination was expressed as a percentage of total seeds per replicate.

To evaluate seed viability, the seed coat was removed, and the zygotic embryo was extracted from seeds collected in 2019, 2021, 2022, 2023, and 2024. The excised embryos were immersed in a 1% 2,3,5-triphenyltetrazolium chloride solution (0.5 g 2,3,5-triphenyltetrazolium chloride + 50 mL PBS) and incubated in Petri dishes for 2 hours in a NOVATECH^®^ incubator at 40°C in the absence of light.

After incubation, embryos were removed from the 2,3,5-triphenyltetrazolium chloride solution, rinsed with distilled water, and placed on moist paper towels for evaluation. Observations were performed using a Leica^®^ EZ4 HD stereomicroscope to assess staining patterns of the zygotic embryos. The evaluated variables included the number of viable and non-viable embryos, as well as viability and vigor percentages based on staining intensity, following the criteria of Hernández-Castro et al. (2021) in *A. angustifolia*. Embryos-stained deep red were classified as vigorous, pale red embryos were considered viable with low vigor, and unstained embryos were classified as non-viable.

For each seed collection year, four replicates of 50 seeds were analyzed. Statistical analysis was performed using a Kruskal–Wallis test followed by Dunn’s multiple comparisons test to identify differences in seed viability among years. Analyses were carried out using R version 4.4.1 (R Core Team, 2024), and the PMCMRplus (Pohlert, 2024) package.

### 2.3 Somatic embryogenesis induction

Seeds were initially washed with 300 µL of TWEEN^®^ 20 and 500 µL of RIDOMIL GOLD^®^ 480 in 250 mL of distilled water under continuous agitation for 1 hour. Following this, under a laminar flow hood, seeds were disinfected in 100 mL of a solution composed of 5% sodium hypochlorite and sterile distilled water in a 30:70 v/v ratio with 150 µL of TWEEN^®^ 20 for 15 minutes. Seeds were then rinsed three times with sterile distilled water to remove residual disinfectant and left in the third rinse container to imbibe for 24 hours. Embryonic axes were extracted from the seeds under a stereomicroscope in aseptic conditions and used as initial explants. Somatic embryogenesis induction was performed using two auxins tested at different concentrations: 0.0, 9.06, 18.12, 27.18, and 36.24 μM of 2,4-dichlorophenoxyacetic acid (2,4-D), and 0.0, 8.28, 16.56, 24.84, and 33.12 μM of 4-amino-3,5,6-trichloro-2-pyridinecarboxylic acid (picloram) following Delgado-Aceves et al. (2021). Each auxin was independently combined with 0.0, 2.22, 3.33, or 4.44 μM of 6-benzylaminopurine (BAP). Embryonic axes were incubated under dark conditions for 40 days at 25 ± 2°C for induction.

Subsequently, the resulting calli were transferred to Petri dishes containing modified semi-solid MS (Murashige and Skoog, 1962) expression medium (with NH_4_NO_3_ reduced to 5 mM), supplemented with 500 mg L^-1^ glutamine and 250 mg L^-1^ casein hydrolysate, and cultured for an additional 60 days, following the protocol described by Delgado-Aceves et al. (2021). During this phase, cultures were exposed to a 16/8 h light/dark photoperiod at a light intensity of 27 mol m^-2^ s^-1^.

A completely randomized factorial design (CRD) was implemented to evaluate the effects of two plant growth regulators on callogenesis in *Agave maximiliana*. The experiment followed a 5 × 4 factorial scheme,involving: 2,4-D and picloram, each tested at five levels and combined with four levels of BAP during the induction phase. Each treatment included five replicates, and each experimental unit consisted of one Petri dish (60 x 15 mm) containing 10 mL of semi-solid medium and one zygotic embryo as the explant. Aligned Rank Transform ANOVA (Wobbrock et al., 2011) were carried out with the *ARTool* package (Kay et al., 2015) to evaluate if there was a difference in the percentage of callus formation among the different hormone concentrations, one for 2,4-D and one for Picloram. Estimated marginal means were obtained using the *emmeans* package (Lenth, 2024), and multiple comparisons were conducted with the *multcompView* package (Graves et al., 2019). Visualization was created with the *ggplot2* package (Wickham, 2016). All of this was done in R version 4.4.1 (R Core Team, 2024).

The number of somatic embryos generated per callus was recorded after 60 days under a stereomicroscope (Leica^®^ EZ4 W) at 10x magnification.

For all treatments, the mean percentage of callus formation was calculated. Friable and embryogenic calli (those showing somatic embryo formation) were selected and subjected to a second induction cycle on expression medium supplemented with 9.06 μM of 2,4-D. The Embryo-Forming Capacity (EFC) index was defined by the equation previously reported by Delgado-Aceves et al. (2021):


EFC=(Average percentage of calli forming SE) (Average number of formed SE)/100

### 2.4 Histological analysis

Additionally, samples of calli and somatic embryos were collected in triplicate for each treatment. Samples were embedded in polyethylene glycol (PEG, molecular weight 1450) at a 1:4 ratio (PEG: deionized water) following the protocol described by Burger and Richter (1991). A rotary microtome was used to obtain 15 µm-thick sections from the PEG-embedded material. Subsequently, a double staining procedure was performed using 0.5% acetocarmine and 0.5% Evans blue in a 1:1 (w/v) ratio, as described by Gupta and Durzan (1987). The same staining protocol was applied to fresh samples. Observations were conducted using a light microscope (Leica^®^ Microsystems EZ4 W).

## 3 Results and discussion

### 3.1 Capsule characterization and germination

**Figure 1:**
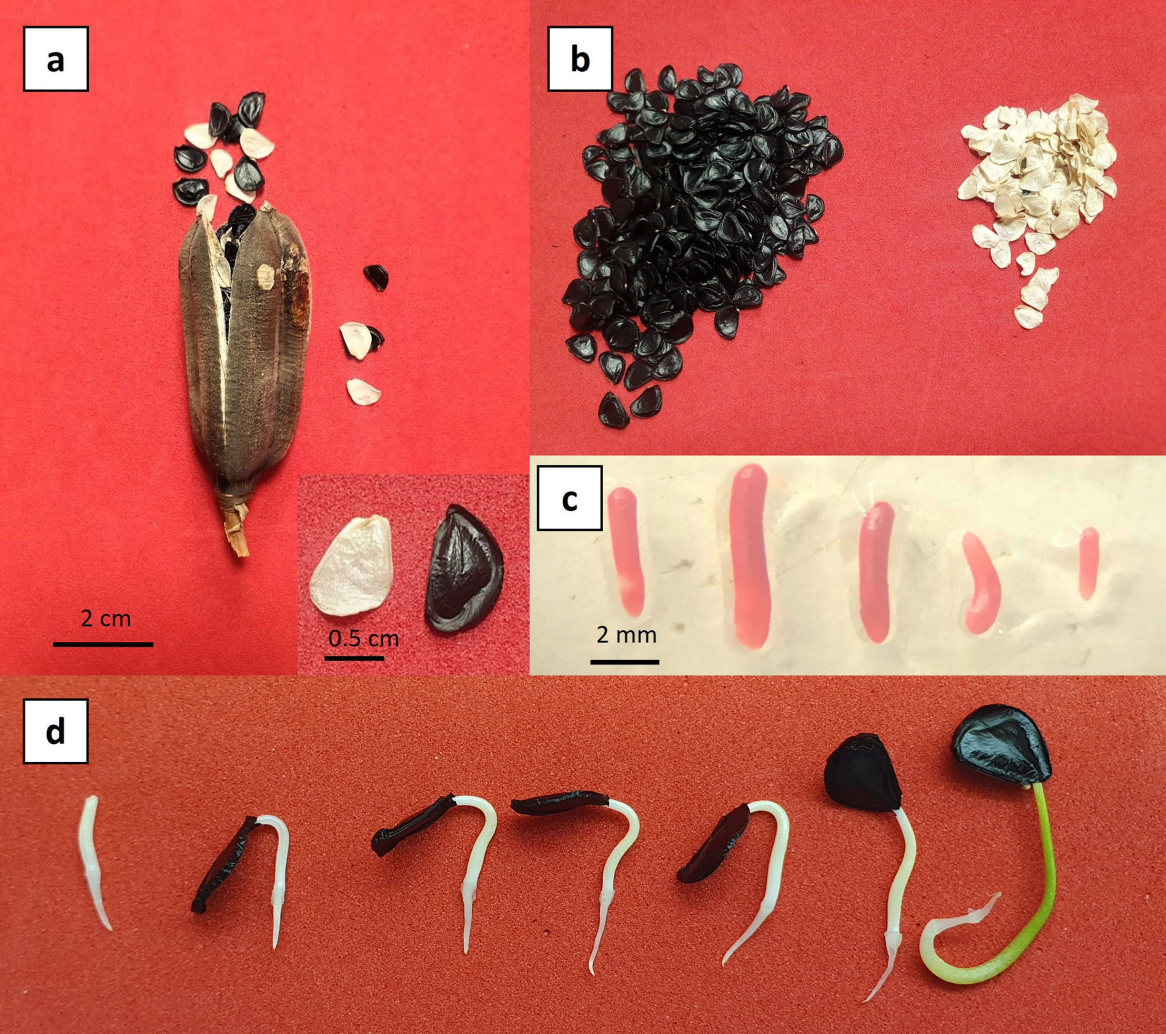
Biological material of *Agave maximiliana*. **(a)** Mature fruit. **(b)** Viable (black) and non-viable (white) seeds. **(c)** Tetrazolium-stained zygotic embryos showing full viability. **(d)** Seed germination.

In this study, the capsules exhibited average dimensions of 6.5 cm in length and 2.6 cm in width. A preliminary seed count per fruit revealed an average of 274 black seeds and 131 white seeds, based on a sample of 10 capsules in 2023 sample (**Figure 1**).

Although the *Agave maximiliana* seeds analyzed in this study were collected from the same geographical region, the results show that the year of collection had a considerable effect on seed viability and germination. While seeds collected in 2023 and 2024 exhibited high germination rates (98.4% ± xxx and 90.0 ± 9.8%, respectively), no germination was observed in samples from 2019, 2021, and 2022, despite being evaluated under similar conditions. These findings suggest that factors associated with the year of collection—such as interannual climatic variability, the physiological status of the mother plants at the time of fruit development, or postharvest handling—can significantly influence seed viability, even within the same collection area. This highlights the importance of considering both environmental and temporal contexts in propagation studies.

Additionally, other factors such as seed size or comparisons across distinct geographical zones should also be considered in future germination studies. For instance, seed size has been shown to influence seedling emergence and development in *A. salmiana* (Vázquez Díaz et al., 2011), although in some cases, such as *A. potatorum*, germination has varied more strongly with collection date and treatment conditions rather than seed size itself (Villavicencio-Gutiérrez et al., 2024). These considerations highlight the need for a multifactorial approach to understanding germination behavior in *Agave* species.

### 3.2 Statistical analysis and viability evaluation

Statistical analyses revealed significant differences in seed viability among the evaluated years (chi-squared = 17.544, df = 4, p-value = 0.001515). Specifically, seeds from 2024 exhibited significantly higher viability compared to those from 2019, 2021, and 2022 (P< 0.05), but not compared to seeds from 2023.

**Figure 2:**
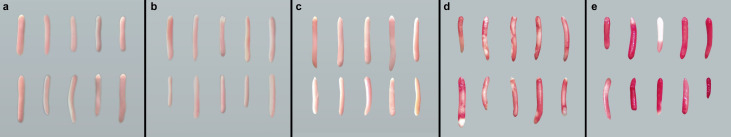
*Agave maximiliana* embryos stained with tetrazolium. **(a)** Embryos from 2019. **(b)** Embryos from 2021. **(c)** Embryos from 2022. **(d)** Embryos from 2023. **(e)** Embryos from 2024.

From a qualitative perspective, although no significant differences were observed in the viability percentages between the 2023 and 2024 seeds, a clear difference in embryo vigor was evident. Embryos from 2024 exhibited a more intense coloration when stained with tetrazolium, in contrast to those from 2023 (**Figure 2**). These results suggest that *A. maximiliana* seeds experience a significant loss of viability over time, to the extent that after two years of storage at room temperature, they become practically unusable.

The viability of biological material plays a crucial role in the success of somatic embryogenesis induction (Ángeles-Vázquez et al., 2023). The viability of zygotic embryos is a critical factor in the successful induction of somatic embryogenesis, as it directly influences the cellular competence of the explant and the subsequent morphogenic response (Ángeles-Vázquez et al., 2023). Viable embryos provide metabolically active, totipotent cells capable of re-entering the embryogenic pathway under appropriate *in vitro* conditions (Merkle and Nairn, 2005). The physiological state of the embryo at the time of excision—particularly its developmental stage, hydration level, and cellular integrity—can markedly affect its responsiveness (Gaj, 2004). Several studies have demonstrated that immature and metabolically active zygotic embryos often exhibit higher embryogenic potential compared to mature or desiccated ones (Jiménez, 2005; Quiroz-Figueroa et al., 2006). Therefore, assessing and ensuring the viability of zygotic embryos prior to culture is essential to optimize somatic embryogenesis protocols, particularly in species with low regenerative capacity or high susceptibility to browning and necrosis.

### 3.3 Comparative analysis of callogenesis induction

The induction of callogenesis in treatments with 2,4-dichlorophenoxyacetic acid (2,4-D) and 6-benzylaminopurine (BAP) did not exhibit statistically significant differences among the combinations tested (p > 0.05), as all treatments were assigned to the same statistical group (“a”). Nonetheless, a positive trend was observed in the estimated percentages of callus formation, ranging from approximately 30% in the control (0 µM 2,4-D + 0 µM BAP) to over 70% in the treatment containing 9.05 µM 2,4-D + 3.33 µM BAP. Although increasing concentrations of 2,4-D (up to 36.20 µM) appeared to promote a gradual enhancement in callogenesis, this trend was not supported by statistical significance. Likewise, the presence or absence of BAP within the range of 2.22–4.44 µM did not yield any meaningful variation in the formation of callus. These results suggest that, while 2,4-D may have a role in promoting callogenesis, its effect under the tested conditions was not sufficient to generate statistically distinguishable responses, and BAP showed no clear additive effect.

In contrast, the response of callogenesis to treatments combining picloram and BAP varied significantly. The control (0 µM picloram + 0 µM BAP) exhibited the lowest callus induction (≈22%) and was statistically different from the remaining treatments. A progressive increase in callogenesis was observed with higher concentrations of picloram, reaching a maximum response of approximately 85% in treatment 3 (16.56 µM picloram + 0 µM BAP). Overall, increasing picloram levels (up to 33.13 µM) were positively associated with enhanced callogenic potential. However, as observed with 2,4-D, the inclusion of BAP (2.22–4.44 µM) did not significantly improve the callogenic response, regardless of the auxin concentration. These findings indicate that picloram is essential for inducing callogenesis in this system, with the optimal response achieved at 16.56 µM, while BAP does not play a significant role in enhancing this effect.

**Figure 3:**
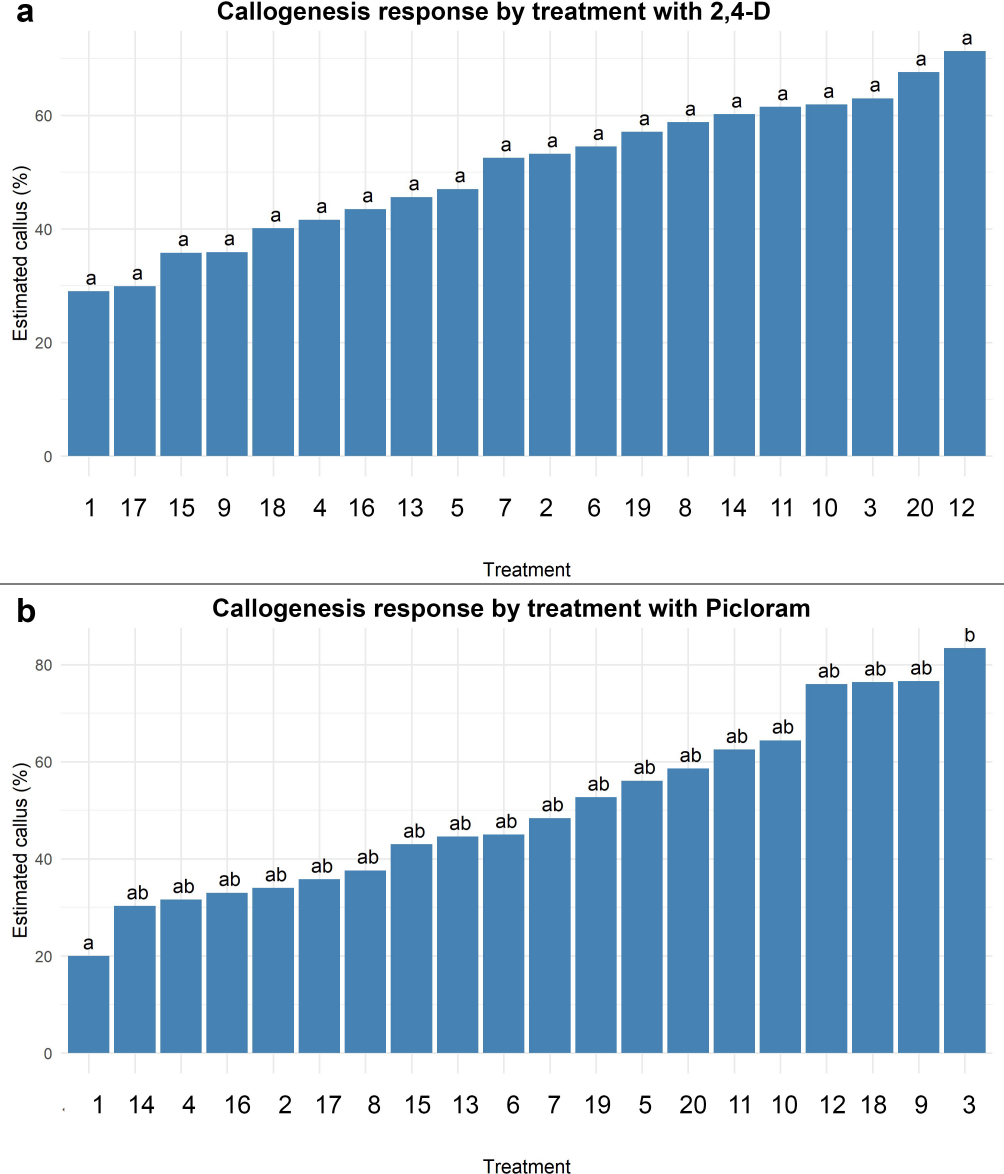
Estimated callogenesis response (%) in *Agave maximiliana* under different combinations of auxin and 6-Benzylaminopurine (BAP). **(a)** Treatments using 2,4-D, **(b)** Treatments using Picloram. Bars represent estimated marginal means (with 95% confidence intervals) from an ART ANOVA model. Treatments are ordered from lowest to highest response. Different letters above bars indicate statistically significant differences between treatments (Tukey-adjusted, p< 0.05).

Supporting these observations, the aligned rank transform (ART) ANOVA revealed a significant effect of 2,4-D concentration on callogenesis (p< 0.001). *Post hoc* comparisons indicated that the only statistically significant differences occurred between treatments lacking 2,4-D and those that included it. In the case of picloram, ART ANOVA revealed significant main effects of both auxin and cytokinin concentrations, as well as a significant interaction between the two factors (p< 0.001 in all cases). Multiple comparisons identified only treatment 3 (16.56 µM picloram + 0 µM BAP) as being significantly different from the control (p = 0.021), positioning it as the most effective treatment, although only marginally superior to other picloram-based combinations (**Figure 3**).

Although the combination of picloram and BAP demonstrated high efficacy in inducing embryogenic calli, it is important to reiterate that such success is ultimately modulated by the genotype of the explant, which governs the cellular response to exogenous regulators. In *Agave* species, differences in endogenous hormone sensitivity, metabolic activity, and chromatin organization across genotypes influence both the rate and quality of somatic embryogenesis (Rodríguez-Garay, 2016; Monja-Mio and Robert, 2016). In this study, the use of *A. maximiliana* zygotic embryos from a single natural population helped control for genotypic variation, yet the observed variability across treatments further supports that embryogenic competence is inherently genotype-dependent, with hormonal combinations acting as modulators rather than primary drivers.

The auxin picloram has been reported as one of the most effective compounds for promoting extensive callus formation in *Agave* species (Monja-Mio and Robert, 2013; Delgado-Aceves et al., 2021). This high callogenic potential supports the generation of a larger number of dedifferentiated cells, which under appropriate conditions may become polarized and initiate embryo formation. However, the genotype and type of callus (particularly friable), among other abiotic factors, play a critical role in determining the embryogenic potential (Ramírez-Mosqueda, 2022).

### 3.4 Auxin efficacy and hormonal synergy growth regulators in somatic embryogenesis

The purpose of testing different concentrations and modes of action of auxins to evaluate their ability to induce callus formation in zygotic embryos was to assess the feasibility of using higher or lower concentrations for optimal protocol standardization. Treatments with 2,4-D and picloram resulted in high callus formation rates, callus formation was virtually absent in treatments containing only BAP (without auxins), confirming that BAP alone does not induce callogenesis. The most effective treatment, which combined 36.24 µM 2,4-D, 33.12 µM picloram, and 4.44 µM BAP respectively, achieved an EFC of 20%, suggesting a possible synergistic effect at higher concentrations of both auxins and cytokinins.

An increasing trend in EFC was observed with higher BAP concentrations, although greater variability in the data was also noted. Importantly, callus formation alone did not guarantee the subsequent formation of somatic embryos, indicating that the factors inducing callogenesis do not necessarily trigger somatic embryogenesis. Some treatments that produced a high percentage of callus yielded an EFC of zero, which may suggest the need to modify the type of auxin or its ratio with BAP (**Table 1**). Notably, treatment 15 (containing 3.33 µM BAP) also stood out, reaching an EFC of 31.92, indicating that this parameter could be associated with indirect regeneration or another embryogenic response.

**Table 1:** Concentrations of 2,4-D and/or picloram on response of eficiency formation capacity (EFC) from zygotic embryos explant of *Agave maximiliana* after 100 d of culture.

Concentration of growth regulators (µM)				Formation calli (%) 2,4-D Picloram		EFC 2,4-D Picloram	
Treatment	2,4-D or Picloram		BAP				
1	0.00	0.00	0.00	0	0	0	0
2	9.06	8.28	0.00	90	92	0	0
3	18.12	16.56	0.00	90	92	6.48	0
4	27.18	24.84	0.00	86	92	0	0
5	36.24	33.12	0.00	78	96	0	0
6	0.00	0.00	2.22	0	0	0	0
7	9.06	8.28	2.22	76	88	0	0
8	18.12	16.56	2.22	68	48	0	0
9	27.18	24.84	2.22	70	98	0	0
10	36.24	33.12	2.22	78	88	0	0
11	0.00	0.00	3.33	0	0	0	0
12	9.06	8.28	3.33	86	92	0.344	0
13	18.12	16.56	3.33	64	60	0	0
14	27.18	24.84	3.33	86	76	0	0
15	36.24	33.12	3.33	50	70	8.8	31.92
16	0.00	0.00	4.44	0	0	0	0
17	9.06	8.28	4.44	68	82	0	0
18	18.12	16.56	4.44	66	84	15.048	0
19	27.18	24.84	4.44	86	94	9.46	0
20	36.24	33.12	4.44	82	82	20.008	0

Indirect somatic embryogenesis induced from zygotic embryos has proven to be a viable strategy, primarily due to the accessibility, quality, and physiological state of the embryos used as explants. Several studies have reported that the efficiency of this process is closely linked to the viability of zygotic embryos, making it a key determinant in the successful induction of embryogenic structures (Arzate-Fernández and Mejía-Franco, 2011; Ángeles-Vázquez et al., 2023). The choice of plant growth regulator must be strategic and based on three key factors: the plant species used, the specific stage of the protocol (induction or maturation), and the production goal—whether to maximize the number of embryos or prioritize their regenerative quality (Jiménez, 2005; Von Arnold, 2008). Making informed decisions in this regard enables the optimization of *in vitro* culture systems and enhances the overall efficiency of plant biofactories.

To better understand the role of auxins in the induction of indirect somatic embryogenesis, a comparative analysis of the behavior of the main auxins used in regeneration processes is presented (**Table 2**). Picloram, a synthetic auxin, has proven to be highly effective in inducing both callogenesis and somatic embryogenesis in various plant species, notable for its high capacity for cellular penetration and its strong potential to trigger dedifferentiation (Machakova et al., 2008). In contrast, 2,4-D, another widely used synthetic auxin, is well known for promoting cell division and maintaining totipotency in *in vitro* cultures, making it a key component in many somatic embryogenesis protocols.

**Table 2:** Comparative analysis onembryogenic behavior of the auxins 2,4-D and Picloram.

Aspect	Picloram	2,4-D (2,4-Dichlorophenoxyacetic acid)	Reference
Type	Synthetic auxin (pyridine-based)	Synthetic auxin (phenoxyacetic-based)	Hayashi, 2021
Main use	Induction of callogenesis and somatic embryogenesis	Induction of cell division and maintenance of totipotency	Von Arnold, 2008; Jayanthi et al., 2015; Monja-Mio and Robert, 2016; Rodríguez-Garay, 2016.
Cellular uptake	High	Moderate	Baur and Bovey, 1970; Sharma and Vanden Born, 1973.
Level of dedifferentiation	Elevated	High, but tends toward greater organization	Delgado-Aceves et al., 2021; Portillo et al., 2007; Reyes-Díaz et al., 2018
Callus formation	Tends to generate abundant callus prior to somatic embryogenesis	Produces less friable callus, favoring more organized structures	Delgado-Aceves et al., 2021; Reyes-Silva et al., 2022
Species-specific efficiency	Some species respond better to Picloram	Useful systems requiring initial induction	Monja-Mio and Robert, 2016; Rodríguez-Sahagún et al., 2011
Gene expression	Regulates embryogenic genes without causing significant oxidative stress	Induces embryogenic genes, but with higher ROS production, which may affect viability	Prigge et al., 2016; Hayashi, 2021
Impact on ROS	Low	High (particularly in leaf explants)	Portillo and Santacruz-Ruvalcaba, 2006; Garcia et al., 2019
Suggested application by stage	Initial induction phase to maximize embryogenic response	Maturation phase to improve embryo quality and organization	Portillo et al., 2007
Practical advantage	Effective in inducing callus and embryos, useful for large-scale production	Produces higher-quality, regenerable embryos	Observed in this study

**Figure 4:**
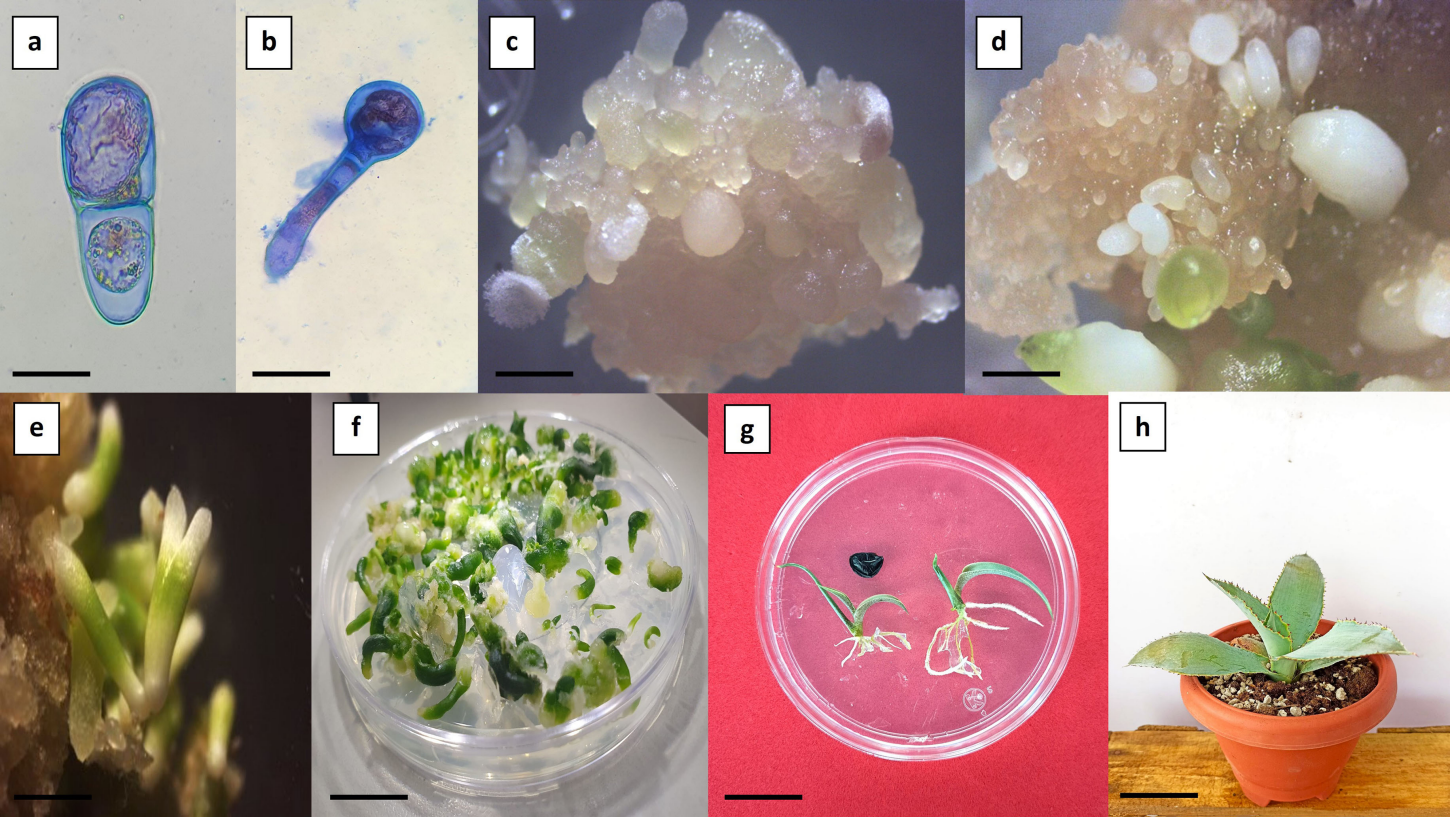
Indirect somatic embryogenesis in *Agave maximiliana*. **(a)** Asymmetric cell division evidenced by differential staining (bar = 25 µm). **(b)** Formation of proembryogenic structures (bar = 100 µm). **(c)** Friable callus with globular-stage somatic embryos (bar = 2 mm). **(d)** Friable callus with coleoptilar-stage somatic embryos (bar = 1.5 mm). **(e)** Presence of somatic embryos with dicot-like morphological characteristics (bar = 3 mm). **(f)** Germination of somatic embryos (bar = 5 mm). **(g)** Conversion and developmental comparison of seedlings: zygotic origin (left) and somatic origin (right) (bar = 1.5 cm). **(h)** Nine-month-old plant derived from somatic embryogenesis (bar = 5 cm).

Auxin responses can be regulated through three main pathways: auxin metabolism, polar auxin transport, and signal transduction. Although synthetic auxins differ structurally, they are generally believed to interact with the TIR1/AFB-Aux/IAA co-receptor complex to activate auxin signaling. However, each synthetic auxin may trigger distinct biological responses due to differences in metabolic stability, polar transport dynamics within tissues, and specific affinities for various auxin receptor complexes (Hayashi, 2021). Indirect somatic embryogenesis was evidenced by asymmetric cell divisions, during which the apical–basal developmental axis of the forming embryo began to be established. Subsequently, the proembryogenic structures gave rise to the formation of the somatic embryo proper in the apical region (stained red) and the suspensor in the basal region (stained blue) (**Figure 4a**). The similarity between zygotic and somatic embryogenesis is further supported by the development of morphologically and functionally equivalent structures as well as by the differentiation of tissues characteristic of the embryo (Rodríguez-Garay, 2016). According to the observations, during the coleoptilar stage, the embryonic axis and radicle are formed independently from the originating callus (**Figures 4b–f**). In the foliar stage, the shoot and root meristems become defined, along with the differentiation of leaf primordia (**Figure 4g**) (Delgado-Aceves et al., 2021).

The conversion rate of germinated somatic embryos into established plantlets reached up to 90% (**Figure 4h**). However, some somatic embryos displayed abnormal development, characterized by amorphous growth and/or arrested radicle formation. These abnormalities are likely associated with somaclonal variation induced by high concentrations of auxins (Garcia et al., 2019). Somatic embryos originate from a single somatic cell, which allows them to retain the genetic fidelity of the donor plants used *in vitro* propagation and conservation (Lambardi et al., 2008). However, the potential for somaclonal variation within the system must be considered.

### 3.5 Histological observations and asynchronous embryo development

**Figure 5:**
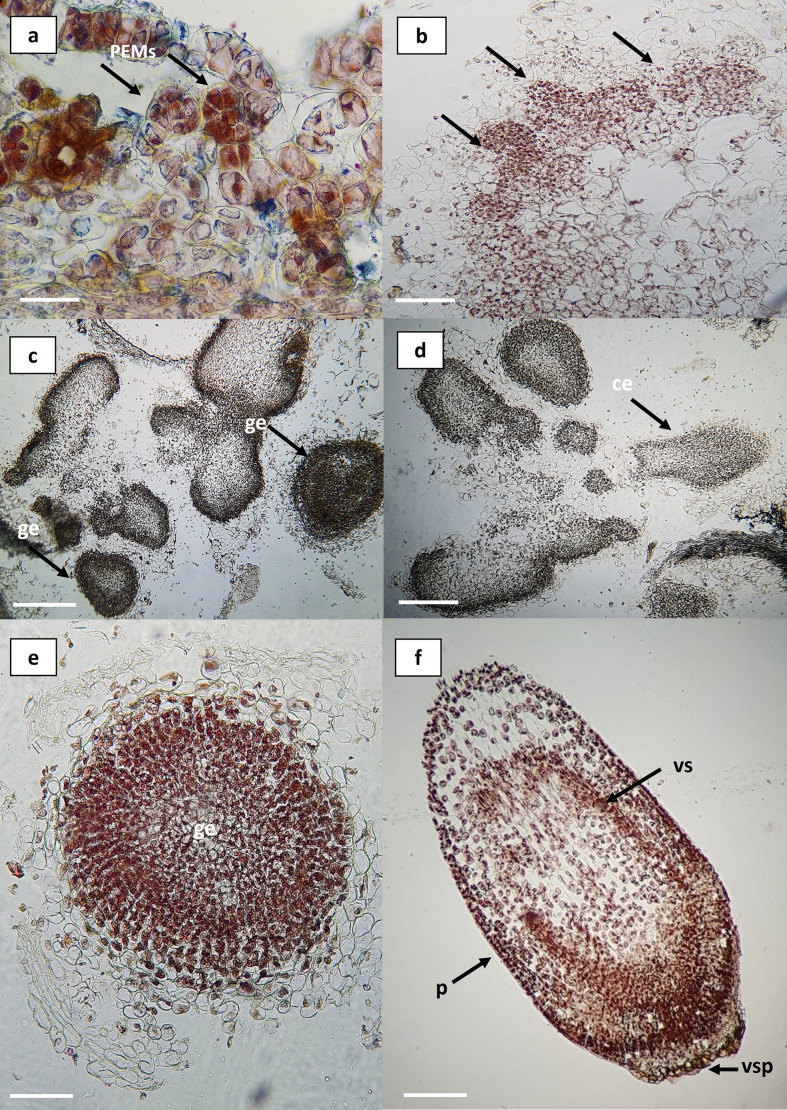
Histological sections of embryogenic callus in *Agave maximiliana*. **(a)** Cluster of pro-embryogenic cells (PEMs) in friable callus (bar = 100 µm). **(b)** Differentiation at the periphery of friable callus (black arrows) (bar = 200 µm). **(c, d)** Organized structures within the callus (bar = 1 mm). **(e)** Somatic embryo at the globular stage (bar = 100 µm). **(f)** Somatic embryo at the coleoptilar stage (bar = 100 µm). PEMs, pro-embryogenic masses; *ge*, globular embryo; *ce*, coleoptilar embryo; *p*, protoderm; *vs*, vascular strands; *vsp*, suspensor vestiges.

Histological observations revealed that the differentiation and reorganization of embryogenic callus occur through a process of progressive cellular independence within the callus itself. It was possible to identify organized structures and characteristic tissues of somatic embryos, including embryos at globular and coleoptilar stages (**Figure 5**). The regeneration system exhibited asynchronous development, allowing the simultaneous observation of multiple embryogenic stages. Initial observations showed clusters of proembryogenic cells (PEMs) (**Figures 5a, b**) which represent a transitional stage toward embryogenesis in the presence of auxins. Within the callus tissue, appropriate hormonal gradients are established, leading to the formation of stem cell niches and their subsequent differentiation (Fehér, 2019). These embryo-competent cells display specific traits such as early activation of the cell division cycle, a more alkaline vacuolar pH, altered auxin metabolism, and non-functional chloroplasts (Zavattieri et al., 2010). The reorganization of the embryogenic callus demonstrated defined cell clusters and boundaries, giving rise to tissues characteristic of somatic embryos (**Figures 5c, d**). The globular and coleoptilar stages observed in this regeneration system (**Figures 5e, f**) are consistent with those reported in *Agave cupreata* (Hernández-Solís et al., 2023), *A. salmiana* (Ángeles-Vázquez et al., 2023), *A. tequilana* ‘Chato’ (Delgado-Aceves et al., 2021), *A. angustifolia* (Arzate-Fernández and Mejía-Franco, 2011), *A. tequilana* ‘Azul’ (Portillo et al., 2007), and *A. sisalana* (Nikam et al., 2003).

Therefore, the combination of picloram with the cytokinin BAP provides an effective method for the induction of indirect somatic embryogenesis.

## 4 Conclusion

This study presents, for the first time, a successful protocol for indirect somatic embryogenesis in *Agave maximiliana* using zygotic embryos as initial explants. The results demonstrate that both 2,4-D and picloram are effective in inducing embryogenic callus formation, with picloram showing higher efficiency and dose-dependent behavior. The optimal combination of auxins and cytokinins (36.24 µM 2,4-D, 33.12 µM picloram, and 4.44 µM BAP) yielded an EFC of up to 20%, while embryo-to-plantlet conversion rates reached 90%. Histological analyses confirmed the asynchronous development of somatic embryos and their structural and functional similarity to zygotic embryos. The progressive loss of seed viability within two years of storage, along with the species’ reproductive limitations, highlights the urgent need for alternative propagation methods. This protocol offers a reliable and scalable biotechnological tool for the conservation, reforestation, and sustainable production of *A. maximiliana*, contributing to the preservation of its genetic diversity and supporting the long-term viability of production systems. Furthermore, integrating this system into agroforestry schemes could support both the conservation and sustainable production of *A. maximiliana* in regions under pressure from Raicilla commercialization.

It is recommended to continue experiments using auxin and cytokinin concentrations near those that promoted somatic embryo formation. Additionally, it is essential to conduct genetic validation studies to ensure clonal stability, as well as to evaluate the agronomic performance of the regenerated plants under field conditions.

## Data Availability

The raw data supporting the conclusions of this article will be made available by the authors, without undue reservation.
